# Reply to “Shifting the emphasis of brain health literacy from individuals to systems to reduce inequalities”

**DOI:** 10.1002/alz.71625

**Published:** 2026-07-01

**Authors:** Sandra Baez

**Affiliations:** ^1^ Departamento de Psicología Universidad de los Andes Bogotá Colombia; ^2^ Global Brain Health Institute (GBHI) Trinity College Dublin Dublin Ireland

1

I thank Daly^1^ for the thoughtful commentary on our recent study examining the relative contributions of sociodemographic factors and Alzheimer's disease (AD) knowledge to modifiable dementia risk.^2^ His interpretation aligns with our central finding that social disadvantage, reflected in socioeconomic status and education, shows a stronger association with behavioral and health‐related risk than AD knowledge.

I agree that these findings have important implications for how brain health literacy is conceptualized. Daly's call to shift emphasis from individual‐level knowledge toward system‐level capacities to reduce inequalities aligns closely with this perspective. Structural determinants, including socioeconomic inequality,^3^ educational opportunities,^4^ and neighborhood conditions,^5^ play a central role in shaping aging^6^, ^7^ and dementia risk across populations. This evidence highlights how broader social conditions shape opportunities for dementia prevention. Importantly, many of the modifiable risk factors examined in our study are not uniformly distributed but vary across regions and socioeconomic contexts,^6^, ^8^ reflecting underlying structural inequalities. This suggests that variation in dementia risk is partly driven by differences in exposure to these risk factors, which are themselves shaped by these conditions.

At the same time, framing individual‐ and system‐level approaches as alternatives may be unhelpful. Knowledge remains a necessary component of behavior change, even if it is not sufficient on its own. I therefore see these approaches as complementary. As Daly^1^ notes, factors beyond individual control play a substantial role in shaping brain health, and an exclusive focus on individual responsibility may overlook these constraints. Interventions that focus primarily on individual behavior may be less effective in disadvantaged contexts^9^ and may inadvertently widen existing inequalities if access to enabling resources is uneven. Knowledge may increase awareness, but its impact depends on whether individuals can translate that knowledge into action within their environments. Our findings support the need for integrated strategies in which knowledge‐based interventions are paired with structural supports that make healthy behaviors feasible.

These considerations have practical implications. Beyond education campaigns, effective approaches may include expanding access to preventive healthcare and screening in underserved regions, improving environments that support physical activity and social engagement, and reducing structural barriers to healthy lifestyles. Integrating dementia risk reduction into primary care and community‐based programs may also increase the likelihood that knowledge will lead to sustained behavioral change.

Education represents a key structural determinant shaped by public policy. Access to high‐quality education is unevenly distributed and closely tied to broader social inequalities. Education is a central protective factor in aging trajectories,^10^ likely through pathways such as cognitive reserve, health literacy, and life‐course opportunities. In particular, education plays a critical role in the development of health literacy, shaping individuals’ ability to access, understand, and use health information. Importantly, lower educational attainment is closely linked to socioeconomic disadvantage,^11^ which may limit access to healthcare, constrain opportunities to engage in health‐promoting behaviors, and reduce the ability to act on health information. Policies that expand equitable access to quality education, particularly in disadvantaged regions, may therefore have long‐term effects on dementia risk by strengthening both health literacy and life‐course opportunities. Integrating health literacy into school curricula may further support lifelong health behaviors, although its impact will depend on whether individuals can act on that knowledge across contexts.

I also welcome Daly's proposal to consider more “literate” health systems. In this context, advancing such an approach will require clearer operationalization and empirical evaluation of how system‐level factors enable or constrain the translation of knowledge into action. Future research should examine how structural conditions, education, and knowledge jointly shape behavior over time, particularly through multilevel interventions that combine educational, clinical, and policy components.

In summary, addressing dementia risk requires moving beyond individual‐level explanations toward approaches that account for the structural conditions shaping exposure to risk. Integrating these levels will be key to achieving effective and equitable prevention.

## CONFLICT OF INTEREST STATEMENT

The author declares no conflicts of interest. Author disclosures are available in the Supporting Information.

## FUNDING INFORMATION

Sandra Baez is supported by Global Brain Health Institute, Alzheimer's Association, Alzheimer's Society UK, Pilot Awards for Global Brain Health Leaders (Grant Number: GBHI ALZ UK‐ 25‐1289623).

## Supporting information




**Supporting Information**: alz71625‐sup‐0001‐ICJME.pdf
